# Consumers' preferences for fresh yam: a focus group study

**DOI:** 10.1002/fsn3.364

**Published:** 2016-04-13

**Authors:** Carla Barlagne, Denis Cornet, Jean‐Marc Blazy, Jean‐Louis Diman, Harry Ozier‐Lafontaine

**Affiliations:** ^1^ UR1321 ASTRO Agrosystèmes tropicauxINRAPetit‐Bourg (Guadeloupe)F‐97170France; ^2^ UMR AGAPCIRADPetit‐Bourg (Guadeloupe)F‐97170France

**Keywords:** Consumer profile, focus groups, food quality, sectorial innovation, the Caribbean, yam *(Dioscorea sp.)*

## Abstract

In West and Central Africa and in the Caribbean, yam is one of the most important sources of carbohydrates and has a great potential to improve food security. The yam production sector is, however, now challenged by the satisfaction of evolving consumers' preferences. Since little is known about consumers' preferences regarding yams' characteristics, product quality, and the drivers of yam purchase, six focus group discussions were conducted (for a total of 31 participants). Among the purchasing criteria, price was considered more important than the others. It was followed by the external damage, the origin, and the size of the tuber. The most frequently cited consumption criteria were the taste, the texture, and color of flesh after cooking. Taste was considered more important than the other criteria. Three consumers' profiles were established reflecting heterogeneity in preferences, especially as concerns the willingness to pay for yam and consumption habits. They were designated as the Hedonistic, the Thrifty and the Flexible. Our results suggest that innovations can be implemented to sustain and stimulate the development of the yam sector in Guadeloupe. Two main development paths were identified. The first path is the valorization of the great existing diversity of yam varieties and the increase in the level of information for consumers about product attributes such as the cooking mode, the origin, and the mode of production. Building a marketing strategy based on the valorization of this diversity can help maintain and preserve yam's agro‐biodiversity and the satisfaction of rapidly evolving consumption habits. The second path is the definition of yam ideotypes that suit consumers' needs. We expect that tailoring the production to consumers' needs will have a positive impact on global food security in the Caribbean region.

## Introduction

Yam (*Dioscorea* spp.) is a major source of food for millions of people in tropical and subtropical areas of the world and especially in West and Central Africa where it contributes to the income and food of more than 60 millions of people (Asiedu and Sartie 2010). In West and Central Africa and in the Caribbean, yam is one of the most important sources of carbohydrates to many people and is considered a food security crop. Global projections for root and tuber crops foresee an increase in its economic importance, through the increase in both the production and demand (Scott et al. 2000; Alexandratos and Bruinsma 2012).

Despite its nutritious value (Bradbury and D Holloway 1988) and its major contribution to the daily calorie intake of the population (Asiedu and Sartie 2010), little is known about consumers preferences regarding yams' characteristics and product quality. However, awareness has been raised that a greater integration of consumers preferences in the design of new crop management systems is necessary if sustainable food systems are to be built (Selfa et al. 2008; Rastoin and Ghersi 2010; Tsolakis et al. 2014). This question is all the more crucial, as rural exodus and fast growing urbanization are changing peoples' way of life and consumption habits. Since rural food production may strongly depends on consumers and end markets in urban areas (Campbell et al. 2009), the yam production sector is therefore challenged by the satisfaction of these evolving consumers' preferences (Hounhouigan et al. 2003; Amegbeto et al. 2008). Taking into account consumers' preferences for fresh yam can have two important implications for building sustainable food sectors. First, at the production level, it may help designing new crop varieties and crop management systems that simultaneously satisfy the needs of the farmers and the consumers' preferences (Hounhouigan et al. 2003; Amegbeto et al. 2008). Second, it may also have implications at the market level for identifying the relevant information about the product and the quality attributes that have to be given to consumers.

The aim of the study presented in this paper is to identify consumer preferences for fresh yam through focus groups. The study is conducted in Guadeloupe, a tropical island in the Caribbean where yam has a strong cultural value (Dulcire 2005) and represents the first food crop (Agreste 2009a; Agreste 2009a). Yam production is however declining, since it was 26,700 tons in 1970 and is now about 7 000 tons. The yam sector in the French West Indies has faced many hurdles that hindered its development: 1) high sensitivity to pests and diseases at the field scale (Ano et al. 2005; Arnolin and Lombion 2005); 2) long‐lasting soil pollution by chlordecone, a persistent pesticide, making root and tuber production conditional to strict controls on the final level of contamination of the harvested organs (Cabidoche and Lesueur‐Jannoyer 2012), and 3) poor organization of the actors within the sector. Guadeloupe relies heavily on imports for 80% of its food supply (Chambre d'Agriculture de la Guadeloupe 2014) and in order to enhance food security, research is conducted to increase the sustainability of the yam sector. Research aimed to target the biotic constraints by selecting varieties resistant to the major diseases (Onyeka et al. 2006). Although resistant and high‐yield varieties have been developed, they have not been appreciated by consumers (AGRESTE 2009a,b). As a consequence, farmers have not adopted them. Therefore, a sound assessment of consumer expectations regarding yam characteristics needed to be performed.

## Material and Methods

### Pilot study

In a pilot study, five local experts were asked to describe the main yam varieties that can be found in Guadeloupe as well as to enumerate purchasing and consumption criteria. Among those five experts were: a cook used to experimenting new yam‐based meals; two geneticists and a technician involved in yam breeding programs; and one agricultural advisor involved in participatory selection of yam testing varieties with farmers. All of them had a deep knowledge and experience of yam and were all yam consumers and purchasers. In total, those experts listed 10 purchasing and four consumption criteria. Purchasing criteria included: cooking time, cooking mode, maturity, shape, variety, freshness, origin, size, price, and external damage. Consumption criteria included: color of flesh after cooking, fibrousness, texture of flesh after cooking, and taste.

### Focus group discussions

We used focus group discussions because they were identified as an efficient method to elicit the drivers of consumer choices and explore new product concepts (van Kleef et al. 2005). Focus group discussions allow access to a wealth of information and insights regarding an issue, providing a platform for interaction between the participants within a limited period of time (Morgan 1997; Kitzinger et al. 2004). They are used in market research and sensory analysis to assess consumers behaviors and perceptions (Lee and Lee 2007; Boquin et al. 2014), their attitudes toward new type of food (Wan et al. 2007; Barrios et al. 2008; Chung et al. 2011) or to define consumers’ preferences regarding product quality (Cardinal et al. 2003). They provide qualitative information at the early stages of product development and are often used prior to laboratory and quantitative consumer tests, like sensory assessment and conjoint analysis (Chung et al. 2011; Boquin et al. 2014). At those early stages, they give access to participants’ perceptions of new products that they describe in their own words and meanings. To this end, they provide useful information for the design of appropriate questionnaire and protocol in this field of research.

## Subjects

Six focus group discussions were conducted that accounted for a total of 31 participants. The discussions lasted one hour, and each group consisted of a maximum of six participants to give people sufficient time to express themselves. The conditions for taking part in the focus groups were to be a yam consumer and purchaser. Participants were recruited at the National Institute for Agronomic Research following an email that was sent to all the employees. Participation to the focus group discussion was volunteer and not paid for. Participants were informed about the conditions for participating in the email. Participants were selected irrespective of socio‐economic profiles. There were 15 women and 16 men; ages ranged between 24 and 58; and household size varied from one to six persons. We ensured participants that the results of the study would remain anonymous and would be used for the sole purpose of this study. Additionally, results of the study were fed back to the participants on two occasions.

The number of focus group discussions to be conducted was determined by the principle of data saturation: we stopped doing focus groups when we could obtain no further insight into the different themes from an additional focus group (Krueger and Casey 2009).

## Procedure

One moderator led the focus group discussions, and one observer kept record of the interactions between participants and of the eventual leadership phenomenon during the discussions. The moderator was trained to do focus group discussions as part of her academic curriculum and had already conducted focus group discussions in previous studies (Barlagne 2006, 2007; unpublished). Additionally, before undertaking the study reported here, she validated the methodology and guide of interview with a consultant in polls and opinions surveys, and attended a focus group discussion led by the consultant. The observer also helped in the logistics generated by the discussions by handling the recording device and the material used as support during the discussions. The moderator used a semistructured interview guide to conduct the discussions and maintained a consistent flow of discussion while allowing the flexibility to discuss the themes raised by participants (Krueger and Casey 2009). The themes addressed in the interview guide were related to consumer behavior, purchasing and consumption criteria, the warranty regarding the origin and the mode of production of yams, and the quality of yams. The moderator followed the usual guidelines and animation techniques (Morgan 1997; Kitzinger et al. 2004; Krueger and Casey 2009)to ensure equal participation of the informants in the discussions and to avoid censoring. In particular, in an introductory phase, participants were informed about the objectives of the focus group discussions, and the moderator insisted on the fact that there were no right or wrong answers. The layout of the interview guide is presented in Table 1.

**Table 1:** Layout of the interview guide

Introduction 1.1 Welcoming and introducing the research team 1.2 Definition of the topic of discussion 1.3 Introduction to the rules of focus groups 1.4 Taping and recording of the focus group
Warm‐up and qualification of the panel of participants 2.1 State your name and describe your experience with yams 2.2 Habits regarding yam purchasing and consumption How often do you eat yams? Where do you buy yams?
Probing questions (yam tubers are used as supporting examples for the discussion) 3.1 Purchasing and consumption criteria What do you think of these yams? Would you purchase them? Why? Why not? What do you think is important at the purchasing stage? Discuss each criterion. What do you think is important at the consumption stage? Discuss each criterion. 3.2 Warranty on the origin and the mode of production Do you care about the mode of production of yams? Why? Why not? Do you care about the origin of yams? Why? Why not? Would you be in favor of a warranty for the origin of yams? For the mode of production? How much would you be willing to pay for it? 3.3 Definition of the quality of yams What do you think is a good quality yam? What do you think is a bad‐quality yam? Can you find good‐quality yams on the market?
Conclusion 4.1 Filling out the questionnaire (individually) Tick the criteria that you think are important at the purchasing and consumption stages. Rank the purchasing and consumption criteria in order of decreasing importance. 4.2 Thanking participants for their involvement and concluding the discussion

Yam tubers were used as supporting examples for the discussions after the results of the pilot study. The tubers were purposely chosen for their contrasting characteristics (variety, size, shape, weight, origin, and mode of production) to provoke participant reactions and stimulate the discussion. Participants could examine and handle the tubers. A description of the different varieties used as supporting examples for the focus group discussions or mentioned by the participants during the discussions is provided in Table 2.

**Table 2:** Perception of YAM varieties by local experts

Local name	Scientific name of the specie	Colour of the flesh after cooking	Size	Fibrosity	Texture of the flesh after cooking	Cooking time	Origin	Taste	Mode of production
*Kabusah*	*Dioscorea alata*	White	Medium	Not Fibrous	Tender	8 to 10 min	Local or imported	Neutral	Conventional
*Anba bon*	*Dioscorea alata*	White to light brown	Medium or big	Fibrous	Tender and pasty	8 to 10 min	Local	Wild	Conventional
*Pas Possible*	*Dioscorea esculenta*	White	Small	Not Fibrous	Tender	8 to 10 min	Local	Slightly sweet and refined	Conventional
*Adon*	*Dioscorea bulbifera*	Grey	Small	Not Fibrous	Firm	20 to 30 min	Local	Bitter	Conventional
*Cousse Couche*	*Dioscorea trifida*	White	Small	Not Fibrous	Tender	8 to 10 min	Local or imported	Slightly sweet and refined	Conventional
*Igname Jaune*	*Dioscorea rotundata*	Yellow	Big	Not Fibrous	Firm	20 to 30 min	Local	Bitter	Conventional
*Grosse caille*	*Dioscorea cayenensis*	White	Medium	Not Fibrous	Firm	20 to 30 min	Local or imported	Neutral	Conventional

The list of 10 purchasing and four consumption criteria that had previously been elicited in the pilot study was presented into a questionnaire to the participants at the end of the focus group discussions. They were first asked to select the criteria they considered important at the purchasing and consumption stages. Each participant was asked to fill out the questionnaire individually. The resulting frequencies of selection gave an understanding of the criteria that count in absolute terms for the participants. Thereafter, they were asked to rank purchasing and consumption criteria from the most important to the least important. This allowed an understanding of the relative importance of the criteria for the participants. The relative importance of the criteria was normalized as expressed in the Data Analysis section. Expressing frequencies and relative importance as percentages allowed a better comparison of the importance of the criteria in the two cases.

Focus group discussions were recorded with a video recorder. This device was important for clarifying the moments when participants designated the yam tubers for each of the activities during the discussions. We completed the focus group discussions with a sociodemographic questionnaire to keep a record of their characteristics.

## Data analysis

### Analysis of the focus group transcripts

The focus group discussions were fully transcribed using video records and field notes. Coding and analysis of the transcripts was performed using the content analysis software NVivo10 (QSR International, Melbourne, Australia). The codes resulted from the original research question and hypothesis and from recurring themes that emerged during the focus group discussions. The data were coded according to 14 main themes that ranged from the appreciation of yams to questions of traceability. The themes and their definition are given in Table 3.

**Table 3:** Themes highlighted in the focus group discussions and their definition

Themes	Definition
Appreciation of yam	Consumers opinion on yam. How much they like it and how much they buy it
Procurement	How consumers get to eat yams. Which marketing chains or gift chains they go through to get yams
Attitude toward price	What consumers think of the price of yam, how they react to it when they intend to buy yam. Type of adjustments they make towards the price
Contamination by pollutants	Whether consumers are suspicious about the contamination of yam by chlordecone or not and how they adapt to it in their purchasing behavior
Knowledge of the product	Whether consumers know different varieties of yam and how they describe them
Conservation	How consumers keep yams and how the different varieties can be kept
Purchasing and consumption criteria	Criteria consumers take into account when they purchase and/or eat yams
Easiness of peeling	Easiness of peeling of the different varieties
Time of consumption	Time of the year or time of the week when people eat yams. Type of celebration they associate yams with
Losses	Losses associated with the peeling of yam or with damages caused by bugs and how consumers related to those losses
Yam quality	Definition of the quality of yams by the participants
Yam status	Perception of yam as a dish and as a cultural food productImportance in consumption habits
Processing	Type of processing of yam by the participants and their opinion about processed yams
Traceability	What do consumers think of traceability and whether they do their purchases in relation to it

Content analysis was performed on all themes. The “Purchasing and Consumption Criteria” theme contained 14 categories that related to the criteria that the participants selected in the questionnaire.

Consumers’ profiles were established following content analysis of the theme “Attitude toward the price of yam”. Transcripts coded under this theme related to participant opinions about the price of yam, their purchasing behavior, and strategies. For each profile identified, we examined the frequencies and relative importance of the purchasing and consumption criteria. We also proceeded to the content analysis of the themes “origin,” “mode of production,” and “traceability” to understand the heterogeneity in perception and interest in those themes. Additionally, queries were run using the “Matrix Coding Query” tool available in the software to examine the intersection between themes or between a theme and participant profiles. This allowed understanding a theme within differentiated categories.

### Analysis of the questionnaire results

The frequency and the relative importance of each criterion were calculated from the questionnaire data. The frequency of a criterion was calculated as the percentage of participants selecting the criterion. The relative importance of a criterion j was ${\overset{¯}{I}}_{J}\;\%$, computed as in the following: ${\overset{¯}{I}}_{J}\;\%=100\frac{\sum_{i=1}^{n}r_{ij}}{\sum_{j=1}^{c}\sum_{i=1}^{n}r_{ij}}$, where *n* is the number of participants, *c* is the number of criteria, and *r*_*ij*_ is the rank attributed to the criteria by participant *i*, ${\overset{¯}{I}}_{J}\%$ varying between 0% (i.e., the least important criteria) and 100% (i.e., the most important criteria).

## Results

### Purchasing and consumption criteria

**Figure 1:**
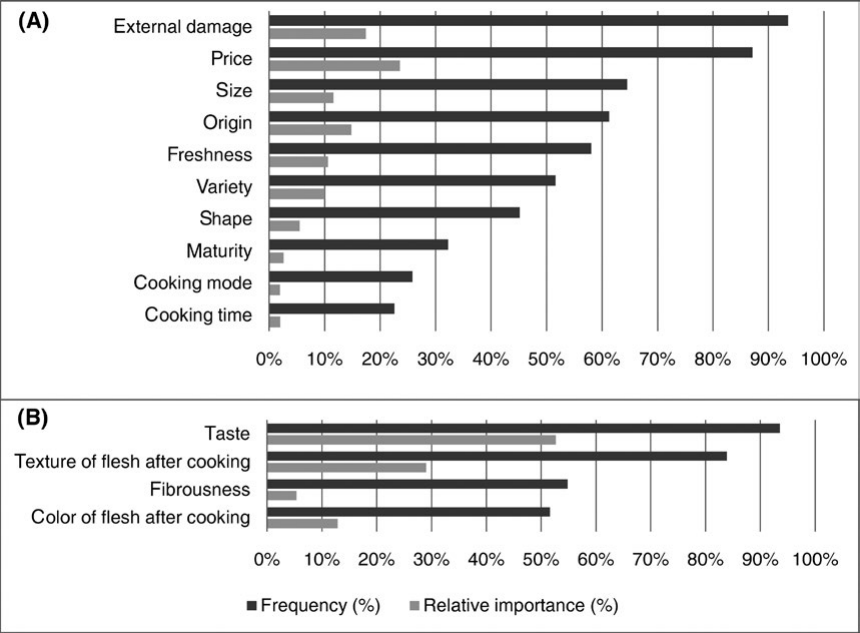
Frequency and relative importance (I_%) of the purchasing (A) and consumption (B) criteria of the participants. Frequency of the criteria represents the number of times the criteria were cited by the participants while Relative Importance represents the ranking of the criteria by the participants

Figure 1 represents the Frequency and Relative Importance (${\overset{¯}{I}}_{J}$%) of the purchasing (1) and consumption (2) criteria of the participants.

Ten purchasing criteria were cited by participants (Fig. 1A). The most frequent criteria were the external damage and price, both of which were selected more than 80% of the time. Four more criteria were cited more than half the time: the size, the origin, the freshness, and the variety. The last criteria considered important were the cooking mode and the cooking time. Among the purchasing criteria, price was considered more important than the others ($\overset{¯}{I}=24\%$). It was followed by the external damage ($\overset{¯}{I}=17\%$), the origin ($\overset{¯}{I}=15\%$), and the size ($\overset{¯}{I}=12\%$). Price and origin were given a higher relative importance than the external damage and the size, respectively, though they were cited fewer times than those criteria.

Four consumption criteria were cited by the participants (Fig. 1B). The most frequent criteria were the taste and the texture of flesh after cooking, both of which were cited more than 80% of the time. The other two criteria, the fibrousness and the color of flesh after cooking, were cited more than 50% of the time. Among the consumption criteria, taste was considered more important than the other criteria with a relative importance ($\overset{¯}{I}=53\%$). The texture of the flesh after cooking was considered the second most important criterion ($\overset{¯}{I}=29\%$). The color of the flesh after cooking was given a higher importance than the fibrousness although it has been cited less.

### Definition of the quality of yams and sensory preferences

Participants defined the quality of yams with a set of eight attributes (or criteria) that referred exclusively to the visual and sensory characteristics. Table 4 presents these attributes and their definition according to the participants.

**Table 4:** Perception of the quality of yams according to the participants (*N* = 31)

Attributes	Good‐quality yam	Bad‐quality yam
Sensory attributes		
Taste	Sweet, bitter, neutral, refined, wild	Undefined
Texture of flesh after cooking	Firm or tender but dense	Lack of consistency; yam that crumbles during cooking; yam that is too firm when cooled
Fibrousness	Absence of fibers	Presence of fibers
Color of flesh after cooking	Plain and clear; white or yellow	Presence of black spots or rotting; brownish; grayish
Visual attributes		
External damage	Healthy external aspect of the tuber	Presence of external damage; presence of insect bites; broken yam
Size	Small, medium, big	Undefined
Shape	Regular that facilitates the peeling	Crooked shape that entails losses when peeling
Maturity	Tuber little wrinkled, big or brown‐colored; presence of buds	Tuber white‐colored

The external damage is the only frequently cited criterion that made consensus (i.e., many people viewed the external damage as important, and all agreed on the expected product). The absence of external damage was associated with a healthy product in the mind of the participants, and a broken yam tuber or one that had external signs of insect bites prompted suspicion of possible rotting inside the tuber. Participants defined the other attributes using a range of values. For example, they expressed their taste preferences using not less than five different words, some of them referring to the usual taste descriptors (sweet, bitter, neutral) and some reflecting particular individual perceptions of the taste (wild and refined). To illustrate their perceptions of the taste of yam, participants referred to specific yam varieties: “the anba bon *(D. alata)* was perceived as having a wild taste” whereas “the taste of the *pas possible (D. esculenta)* was perceived as refined”. The attribute “wild” recalled a woody taste while the attribute “refined” echoed to a sensation of delicacy.

Preferences for the texture were firm or tender, the common requirement being a good density of the piece of yam. Participants disliked a piece of yam that crumbles in the pan during cooking. Two yam varieties were specifically mentioned as having a firm texture: the *igname jaune*, otherwise known as the *yellow yam (D. cayenensis)*, and the *grosse caille*, otherwise known as the *white yam (D.rotundata)*. Participants mentioned that these two varieties were suitable for consumption when freshly cooked but that they tended to become too hard to eat (therefore not suitable for consumption) when cooled. Additionally, the participants established the suitability of the different varieties for different modes of cooking according to texture. Thus, they stated that firm varieties were suitable for fries or gratin, while the tender varieties were more suitable for puree. They considered that both types of texture were suitable for boiling, but that the cooking time had to be adjusted to the type of texture (longer for the firm varieties). In terms of the size, the participants preferred to buy yam tubers suitable for only one meal, in general lunch. They did not eat yams for the evening meal nor on the following day because they believed that yams did not keep well.

### Consumer profiles

**Figure 2:**
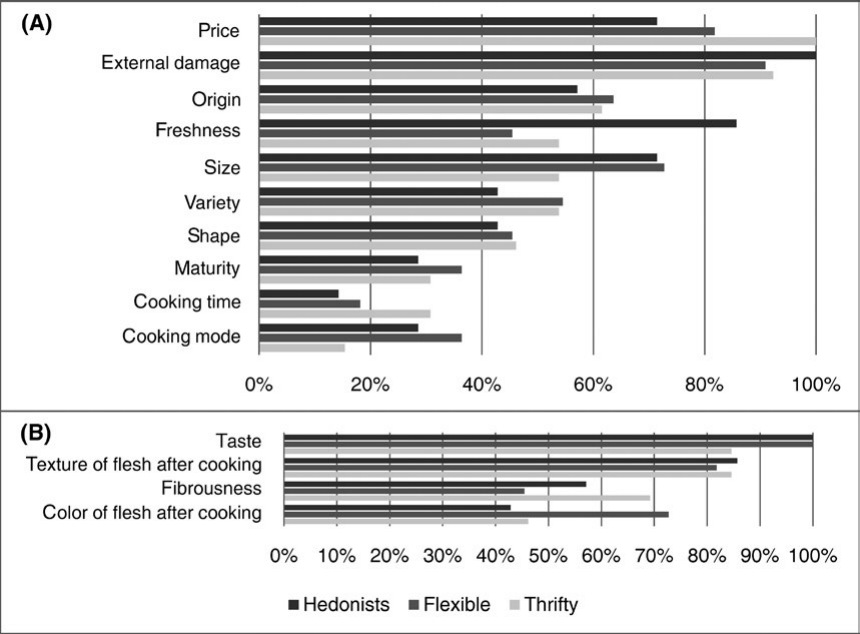
Frequency of the purchasing (A) and consumption (B) criteria according to the participant profiles

**Figure 3:**
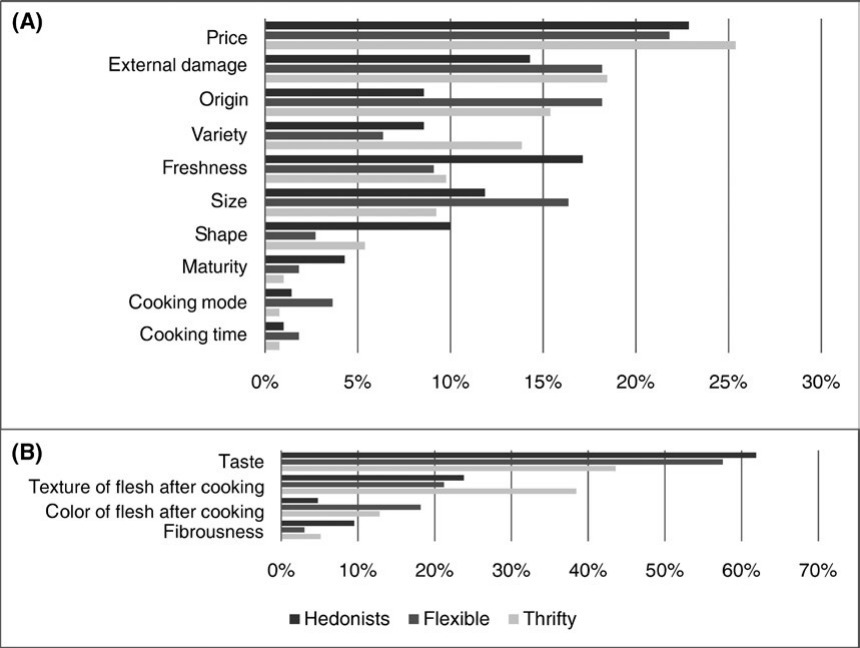
Relative importance of the purchasing (A) and consumption (B) criteria according to the participant profiles

Based on purchasing and consumption criteria, three main profiles emerged among the participants. The first two profiles were distinct: the participants were either Hedonists (23% of the participants) or Thrifty people (42% of the participants). Participants in the third profile had a mixed and changing attitude. We designated this group as Flexible (35% of the participants).We would like to draw attention to the fact that the proportions of different profiles are only indicative of the present sample and that they, by no means, intend to be representative of the global population of yam consumers. They simply reveal that different consumers’ profiles do exist which are relevant in terms of market segmentation. Figure 2 represents the Frequency of the purchasing (Fig. 2A) and consumption (Fig. 2B) criteria for the different profiles of the participants, while Figure 3 represents the Relative Importance of the purchasing (Fig. 3A) and consumption (Fig. 3B) criteria according to the different profiles of the participants.

#### The Hedonists (23% of the participants)

Except for the first criteria (external damage) and the last three criteria (maturity, cooking mode and cooking time), the distribution of the frequencies of the purchasing criteria differed in this group from that of the whole sample. The external damage and the origin were cited more frequently than the price and the shape, respectively, but they were given a lower relative importance (${\overset{¯}{I}}_{J}\;\%$). As for the other criteria, the ${\overset{¯}{I}}_{J}$% they were given by the participants reflected their frequencies (i.e., the criteria were ranked in the same order after their relative importance as well as their frequencies).

Considering the ${\overset{¯}{I}}_{J}$% of the purchasing criteria, the Hedonists placed the emphasis on the price first ($\overset{¯}{I}=23\%$), then on the freshness ($\overset{¯}{I}=17\%$), the external damage ($\overset{¯}{I}=14\%$), and the size ($\overset{¯}{I}=12\%$). As for the consumption criteria, the distribution of the frequencies was similar to that of the whole sample. In spite of the fact that the relative importance of the consumption criteria reflected their frequencies, the Hedonists emphasized the taste over the other criteria ($\overset{¯}{I}=62\%$).

The Hedonists found that local yams were expensive, particularly when out of the production season, but they declared that the price had no importance. “Very often, the price does not mean anything… I mean… from the moment I have decided that I want to eat yam, the price does not interest me anymore”. “If I go somewhere and I buy yams and I find them good, then I'll go back to buy some more and I will not pay attention to the price”. They would pay a higher price for yams in four cases: 1) the quality of the yam is perceived to be better, 2) a willingness to eat yams from organic farmers, 3) a commitment to sustaining local farmers “I buy from guadeloupean farmers and I probably pay more for it. I don't ask myself questions about it. I has never been a problem for me and I think it's good to buy from local farmers” and/or 4) greater purchasing power “Yam is not that expensive still…at 3 to 4 €/Kg, it is not a luxury product. Sometimes, you will buy tomatoes at 7€/Kg. Then it starts to be expensive but as long as it is 3 to 4 €/Kg, it does not seem expensive for me. It's what you cook yam with, that makes it expensive. It might be the fish that you cook alongside that is expensive”. The participants were aware of the price hierarchy that exists between the yam species on the Guadeloupean market, and they justified this by the fact that they thought that some of them had superior sensory characteristics. “It's true that if there is some *cousse‐couche (D. trifida)*, then I will buy it. It's priceless. Of course it depends on the quantity, but if it's the *cousse‐couche,* I'll do it”. This highlights the importance of putting forward sensory characteristics when marketing yams.

This group expressed suspicion about yam contamination by chlordecone and would purchase yams outside the periphery of the contaminated area. They expressed the desire to eat yams produced within low‐input cropping systems and were favorable toward warranties verifying the approach. In terms of the origin, this profile did not appear to be very concerned about the origin of the yams, and this criterion ranked only ninth compared to the other criteria after its relative importance. Participants of this group purchased yams in short marketing chains, that is at farms and stalls alongside the road (73% of the informants within that category), because they were viewed as strongly representative of the traditional aspects of yams.

#### The Flexible (35% of the participants)

In this group, the external damage was cited more frequently than the price and the origin, but it was given a lower relative importance (${\overset{¯}{I}}_{J}\;\%$) than the price and an equal relative importance (${\overset{¯}{I}}_{J}\%$) to the origin. In the same way, variety and shape were cited more frequently than the freshness and cooking mode, respectively, but were given a lower (${\overset{¯}{I}}_{J}\%$). Considering the (${\overset{¯}{I}}_{J}\%$) of the purchasing criteria, the Flexible placed the emphasis on the price first ($\overset{¯}{I}=22\%$), then on the external damage and the origin ($\overset{¯}{I}=18\%$) and the size ($\overset{¯}{I}=16\%$).

As concerns the consumption criteria, the distribution of the frequencies differed from that of the whole sample for the color of the flesh after cooking and fibrousness. The relative importance of the consumption criteria reflected their frequencies, but the Flexible place more emphasis on the taste compared to the other criteria ($\overset{¯}{I}=58\%$).

This group was willing to pay between 2.50 €/Kg and 3.50 €/Kg because they thought that the sensory characteristics of the yams would be worth the price and because they believed that local farmers set fair prices. “In direct sale, I don't ask myself too many questions about the price because I trust the farmer; he knows all the production process and all he invested in the production… I trust him”. Sometimes, they would even pay 4.00 €/Kg for specific varieties which are more expensive on the market. “If it's yellow yam, I know that the price is around 3.50–4.0 €/Kg. If I want to eat those yams, then, yes, I'll pay that price”. They adjust the quantity of yams purchased in the cases of expensive market prices or the frequency of purchase: “If it is very expensive, then I do not buy a lot, and I will cook something else along with it”. “If it is expensive, well… I will buy some now, but I won't next time”. Occasionally, they would purchase imported yams as a lower priced substitute. They also purchased them when no local yams were available on the market. Nevertheless, they thought that imported yams were of a lesser sensory quality than the same local variety, and they considered the local yams purchased in short marketing chains to be to all intents and purposes organic. Participants within that group expressed a strong conviction to eat local yams and thought that three situations would justify a higher selling price of yams: 1) supporting local farmers, 2) compliance with higher quality standards through, for example, organic certification “Organic farmers (…) I prefer to pay more but to buy from them because at least, I know that there is traceability,” and 3) the improved sensory characteristics of the yam varieties. They declared to be in favor of the warranty that certified the yams were produced with less inputs and were ready to pay an additional 1.00 or 2.00 €./Kg to eat organic yams, especially for their children. This group was suspicious about yam contamination by the chlordecone and would purchase yams outside the periphery of the contaminated area. They equally purchased yams in short or diversified and long marketing chains, the latter mainly for the sake of convenience.

#### The Thrifty (42% of the participants)

Considering the (${\overset{¯}{I}}_{J}\%$) of the purchasing criteria, the Flexible placed the emphasis on the price first ($\overset{¯}{I}=25\%$), then on the external damage ($\overset{¯}{I}$), the origin ($\overset{¯}{I}=15\%$), and the variety ($\overset{¯}{I}=14\%$).

As for the consumption criteria, the distribution of their frequencies differed from that of the whole sample aside from the texture. The relative importance ($\overset{¯}{I}$ %) of the consumption criteria show that they placed the emphasis on both the taste ($\overset{¯}{I}=44\%$) and the texture of the flesh after cooking ($\overset{¯}{I}=38\%$).

This profile of participants thought that a convenient price for yams was between 1.50 and 2.50 €/Kg, and they declared that they bought yams within this price range, “2.50 €/Kg is a maximum. I consider that I cannot live here and pay more than 2.50 €/Kg for yam (…) I rely all the more on this principle since I consider that I could grow some myself”. Occasionally, they would concede to paying a higher price for a different variety when highly motivated by its sensory characteristics. This profile tended to consider yams a basic staple product comparable to rice or other starchy products used as substitutes when the market price of yam rises. “The price… there is a maximum when psychologically I do not purchase like 2.00 €/Kg. Above, it's excessive. I always make analogies between a yam‐based and a rice‐based meal, and it costs me 5 to 10 times more per kilogram for a meal when I purchase yams”. They would also use imported yams as a substitute in case of expensive prices for local yams, despite imported yams being viewed as of a lesser quality. “At this time of year [when yam is expensive], people just buy yam from Costa Rica [imported yam] which is much more affordable”. As a consequence, this profile would increase the consumption of yams during the production season, and some participants would even choose to store yams purchased at a good price during the season to have for future consumption. “I am not ready to pay more than 2.50–3.00 €/Kg, because I know that, at some point, in December, the price will go decrease up to 1.50 €/Kg and then I can store some since you can keep it”. This group was concerned with the origin and the mode of production of local yams, but argued that they would purchase them from farmers they knew. They insisted on the importance of buying from local farmers (69% of the participants purchased yams in short marketing chains) who “would use few if any inputs”. Provided with this source, participants would be sure of both the origin and the mode of production of the yams, and they did not express willingness to have an additional warranty regarding the origin and the mode of production.

## Discussion

With regard to the percentage of dry matter (25–33%), a kilogram of yam is three to four times more expensive than a kilogram of rice or pasta; nevertheless, consumers sometimes prefer to keep buying yams. This indicates that there are prospects for the development of the yam sector in Guadeloupe. Market differentiation based on the sensory and cooking mode diversity of yam species and varieties, the development of new yam ideotypes, and also the provision of information about the credence attributes of yam to consumers, are promising opportunities.

### Valorizing the sensory and cooking mode diversity of yams varieties

While consumers always expect quality from a product, this term was not clearly defined for yam in the literature, making it unclear as to what to focus on. In this study, we made clear what quality meant for yam consumers. Here, quality first appealed to the senses of the participants, and it referred to a wide range of attributes of yams. They also described the attributes with a range of values that reflected the heterogeneity of their preferences. Participants defined the quality of yams with several sensory and visual attributes: taste preferences (sweet, bitter, neutral), particular individual perceptions of the taste (refined, wild), an external aspect of the tuber free of damages and a small or medium size of tubers (depending on family size). Yam texture also plays an important role, for example, the firm varieties were suitable for fries or gratin, while the tender varieties were more suitable for puree. Both types of texture were suitable for boiling, but the cooking time had to be adjusted to the type of texture (longer for the firm varieties). This clearly shows the importance of the cooking mode for defining yam quality.

Because quality did not mean one standardized type of yam, it directly reflected the diversity of the yam species and varieties present in Guadeloupe, and this advocates for the valorization of that diversity on the marketing side. However, while participants mentioned the yam varieties to illustrate the specific desired traits or cooking modes, some of them were wrong in identifying the varieties used as supporting examples for the discussion. More generally, few people in Guadeloupe are aware of the diversity of yams. Therefore, improving the level of information available to the consumers might help them make choices that better suit their needs and so motivate them to purchase and eat more yams. For example, as it is made for potatoes, packaging yams and giving information regarding the suitability of the different varieties for different cooking modes (i.e., mashed, fried or boiled) would help consumers to make informed choices. As a consequence, information about the diversity of yams could also result in increased demand for yams. It appears as a promising vector of market differentiation that could help sustain and develop the sector. This can be considered an advantage because a wide range of consumer preferences offers flexibility in terms of the choice of yam varieties from the point of view of the farmer. Building a marketing strategy based on the valorization of this diversity can help maintain and preserve yam's agrobiodiversity and satisfy rapidly evolving consuming habits.

### Increasing consumers' level of information about credence attributes

The different types of consumers that were highlighted in our study reflect consumer segmentation in the market and lay the foundation for addressing product differentiation in the yam sector. All the profiles considered the price as important, but they would neither be willing to pay the same price for yams given a set of attributes nor to pay the same premium to benefit from additional attributes such as warranties. Indeed, all the profiles would consider attributes such as support for local farmers, organic farming, or a lower use of inputs to produce yam to be desirable; however, only the Hedonist and the Flexible would be willing to pay for these attributes and would like to have a warranty for them. The Thrifty did not need a third party warranty because they already had an ensured intrinsic warranty as a consequence of purchasing yams from farmers they knew. Regarding the risk of pesticide contamination, it has to be noted that all the profiles had a risk avoidance strategy: they bought yams outside the contaminated area to ensure that the yams were free of contamination.

Loureiro and Hine (2002) found that sociodemographic characteristics affect consumer willingness to pay for potato attributes such as the local origin, the organic mode of production, and the absence of GMOs. The comparison of the willingness to pay for the different attributes helped them to identify the best niche market for potato growers. Similarly, the marketing of yams could also be improved by the provision of information about the credence attributes that would add value to the product and give increased guarantees for the product to consumers. In this case, we would need to assess if the added value benefits the farmers on the other side of the food supply chain. Indeed, as differentiation and labeling stand on the compliance to specifications, we have to ensure that the additional cost generated is covered by the price premium that consumers said they would be willing to pay. Laboratory experiments give a good indication for what happens in real life (Lusk and Fox 2003; Levitt and List 2007). Because the premiums in our study were declarative and might not properly reflect the real price consumers would pay for new products, this real price should be determined by the implementation of laboratory experiments.

### Toward the definition of new yam ideotypes

Measuring the value of both consumption and purchasing attributes in the Guadeloupean context, as well as understanding consumer preferences using sensory data, can enable the guidance of breeding programs toward the definition of yam ideotypes, tailored to this well‐defined and heterogeneous needs.

Our results indicated that all purchasing and consumption criteria were cited by the participants, but the relative importance they gave to the criteria helped to understand the one they prioritized (the four purchasing criteria: price, external damage, origin, and size; and the two consumption criteria: taste and texture). These attributes are of four different types: sensory (taste, texture), visual (external damage, size), economic (price), and credence (origin). Our results are consistent with the few reported studies, since Aidoo (2009) has shown that majority of yam consumers in Ghanaian urban communities preferred white yam to yellow and water yams, and the most important reason for their preference was taste. Amegbeto et al. (2008), by estimating market demand for fresh yam characteristics using contingent valuation in Togo, showed that producers should focus on small size, low weight, and conical‐shaped tubers. Just like in Guadeloupe, cooking characteristics and esthetic qualities are preferred by urban consumers. Hounhouigan et al. (2003) have shown that culinary and organoleptic characteristics preferred by urban consumers in Benin are texture after cooking, taste, color and digestibility.

Some differences occur across the three profiles as to the composition of this minimum set of attributes. The Hedonists focused on sensory and visual attributes, while the Flexible and the Thrifty focused on the sensory and visual attributes as well as on the credence attribute (origin). The reason for which the Hedonists did not emphasize the origin to a great degree is most likely because certain varieties viewed as having a very high sensory quality can only be purchased as an imported staple today (e.g., cousse‐couche). Additionally, the Thrifty emphasized two sensory attributes (i.e., taste and texture) contrary to the two other profiles.

As consumers intended to maximize their utility across a set of product attributes, they had to make trade‐offs between them in relation to the price. Our results revealed the importance of the price, origin, taste, and texture as drivers of consumer choice. This is in accordance with the literature on consumer values (Lusk and Briggeman 2009) and specific to yam (Amegbeto et al. 2008). As the first step in the research process, our study revealed the trade‐off that consumers made between attributes. Amegbeto et al. (2008) has measured the value of fresh yam characteristics, but he has concentrated on the attributes at the purchasing stage. Other studies have used sensory data alone or in combination with value elicitation methods to understand consumer preferences (Lee and Lee 2008; Combris et al. 2009; Chung et al. 2011).

## Conclusion

Because yam research programs are generally production driven and often lack the consumer perspective, we conducted a demand‐driven study to identify the levers of action for the development of the yam supply chain. We organized in Guadeloupe six focus group discussions accounting for a total of 31 participants to understand the drivers of yam purchasing and consumption. Participants considered the price, origin, taste, texture, external damage, and size as important yam attributes. They defined a good‐quality yam as a regularly shaped tuber with a range of sensory attributes that revealed the diversity of their expectations and preferences. Three profiles of participants were identified, each of which were characterized by different consumer behaviors, suggesting that different yam varieties can answer different needs. Therefore, our results suggest that innovations can be implemented to sustain and stimulate the development of the yam sector in Guadeloupe. Two main development paths were identified. The first path is the valorization of the great existing diversity of yam varieties and the increase in the level of information for consumers about experience attributes such as the cooking mode and credence attributes such as the origin and the mode of production. The second path is the definition of the yam ideotypes that suit consumer needs. The next step of the research process would be to analyze deeper consumers' preferences through the implementation of sensory characterization of yam varieties and laboratory experiments. It would allow measuring quantitatively the sensory characteristics of each yam variety, the sensory and cooking preferences of consumers, and the price they would be willing to pay for the different attributes of yams. This work suggests the detailed investigation of how to best match consumer expectations with the production capacity of yam farmers.

## Conflict of Interest

None declared.
